# Renoprotective Effects of Luteolin: Therapeutic Potential for COVID-19-Associated Acute Kidney Injuries

**DOI:** 10.3390/biom12111544

**Published:** 2022-10-23

**Authors:** Lúcio Ricardo Leite Diniz, Hatem A. Elshabrawy, Marilia Trindade S. Souza, Allana Brunna Sucupira Duarte, Nikhil Madhav, Damião Pergentino de Sousa

**Affiliations:** 1 National Institute of the Semiarid, Campina Grande 58434-700, Brazil; 2 Department of Molecular and Cellular Biology, College of Osteopathic Medicine, Sam Houston State University, Conroe, TX 77304, USA; 3 Department of Pharmacy, Federal University of Sergipe, São Cristóvão 49100-000, Brazil; 4 Department of Pharmaceutical Sciences, Federal University of Paraíba, João Pessoa 58051-970, Brazil; 5 College of Osteopathic Medicine, Sam Houston State University, Conroe, TX 77304, USA

**Keywords:** SARS-CoV-2, coronavirus, renal disease, nephrotoxicity, renoprotective effect, flavonoids, natural products, medicinal plant

## Abstract

Acute kidney injury (AKI) has been increasingly reported in critically-ill COVID-19 patients. Moreover, there was significant positive correlation between COVID-19 deaths and renal disorders in hospitalized COVID-19 patients with underlying comorbidities who required renal replacement therapy. It has suggested that death in COVID-19 patients with AKI is 3-fold higher than in COVID-19 patients without AKI. The pathophysiology of COVID-19-associated AKI could be attributed to unspecific mechanisms, as well as COVID-19-specific mechanisms such as direct cellular injury, an imbalanced renin-angiotensin-aldosterone system, pro-inflammatory cytokines elicited by the viral infection and thrombotic events. To date, there is no specific treatment for COVID-19 and its associated AKI. Luteolin is a natural compound with multiple pharmacological activities, including anticoronavirus, as well as renoprotective activities against kidney injury induced by sepsis, renal ischemia and diverse nephrotoxic agents. Therefore, in this review, we mechanistically discuss the anti-SARS-CoV-2 and renoprotective activities of luteolin, which highlight its therapeutic potential in COVID-19-AKI patients.

## 1. Introduction

Coronaviruses (CoVs) are positive single-stranded (+ss) RNA viruses that have long been described as causative agents of diseases in mammals and birds [1,2]. Since 2002, three human coronaviruses (HCoVs) were associated with severe respiratory disease outbreaks that resulted in a large number of cases and deaths in several countries [3,4]. These three HCoVs include Severe Acute Respiratory Syndrome-CoV (SARS-CoV), which was discovered in China in 2002–2003 [5], followed by Middle East Respiratory Syndrome-CoV (MERS-CoV), first identified in Saudi Arabia in 2012 [6], and lastly the novel SARS-CoV-2 that caused the Coronavirus Disease 2019 (COVID-19) pandemic. SARS-CoV-2 was first reported in Wuhan, China in December 2019, and has resulted in millions of deaths worldwide [4,7,8].

COVID-19 patients primarily present with respiratory manifestations due to infection of alveolar epithelial cells, which highly express angiotensin converting enzyme 2 (ACE2), the identified receptor for SARS-CoV-2 [9]. SARS-CoV-2 binds to ACE2 using the receptor binding domain (RBD) of the spike (S) protein on the viral surface [10]. Following the binding to ACE2, successful viral entry into alveolar epithelial cells requires processing of S protein into S1 and S2 domains by cellular proteases such as the membrane serine protease TMPRSS2 and endosomal cathepsin L [9,11]. The cleavage of S protein is followed by the fusion of a viral envelope with the cellular membrane and the delivery of viral RNA into the cytoplasm [9]. Once in the cytoplasm, the 5′ end of SARS-CoV-2 RNA is translated into two polyproteins, pp1a and pp1ab, which are then processed by two viral proteases, the main protease (Mpro or 3CLpro) and papain-like protease (PLpro) [12]. The products of pp1a and pp1ab processing are 16 nonstructural proteins (NSP1-16) that are important for viral replication, including the RNA-dependent-RNA polymerase (RdRp or NSP12) and other viral proteins such as NSP15 (endoribonuclease) and NSP13 (helicase) [12]. The rest of the genome is translated into other nonstructural proteins and structural proteins (S), membrane (M), envelope (E) and nucleocapsid (N) proteins). Following genome replication, these four structural proteins assemble with viral RNA into new viral particles that are released to infect other cells [12].

In the last three years, the global medical community has devoted intense efforts to control the COVID-19 pandemic, which has resulted in over five million deaths around the world [7,8]. In addition to pulmonary manifestations, various studies have shown that COVID-19 patients—especially patients with underlying comorbidities—may present with acute kidney injury (AKI), and that AKI is strongly associated with high mortality of critically-ill COVID-19 patients, particularly if renal replacement therapy (RRT) is required [13,14]. Studies have reported that over a quarter of COVID-19 patients develop AKI during a hospital stay, and the in-hospital mortality rate might vary from 60 to 100%, depending on the AKI stage, presence of other comorbidities, disease severity, medication use, need of RRT and other clinical issues [15,16]. According to Legrand et al. (2021), COVID-19-associated kidney injuries show low molecular weight proteinuria, while Fanconi syndrome and histological findings point towards tubular injury. Furthermore, regional inflammation, endothelial injury and renal microthrombi might be found in COVID-19-hospitalized patients [16].

Although anti-inflammatory drugs seem to limit the development of severe AKI in patients with COVID-19, the short- and long-term morbidities and deaths associated with AKI in COVID-19 patients necessitate the identification of effective renoprotective drugs [17,18,19].

Medicinal plants are rich sources of natural compounds, which have long been studied for their pharmacological activities against cancer, inflammation, cardiovascular and neurodegenerative disorders [20,21]. Flavonoids are among the most-studied plant-derived bioactive compounds, with important functions in plant growth, development, propagation and protection against abiotic and biotic stresses [22]. Flavonoids have demonstrated a wide range of pharmacological activities such as anticoagulant (e.g., Isorhamnetin), anti-inflammatory (e.g., Quercetin), anti-cancer (e.g., Catechins), anti-microbial (e.g., Myricetin) and anti-depressant (e.g., Orientin) activities [23,24,25,26,27]. The majority of pharmacological activities of flavonoids is attributed to their anti-oxidant properties due to the presence of phenolic rings, which promote the electron donation and hydrogen atom transfer to free radicals, acting as free radical scavengers [28,29]. Moreover, flavonoids have been reported to reduce oxidative stress—a critical factor in the genesis and progression of multiple pathologies—by promoting an increase in the levels of anti-oxidant enzymes (SOD, CAT and GPx) and/or reduction in lipid peroxidation [30].

**Figure 1:**
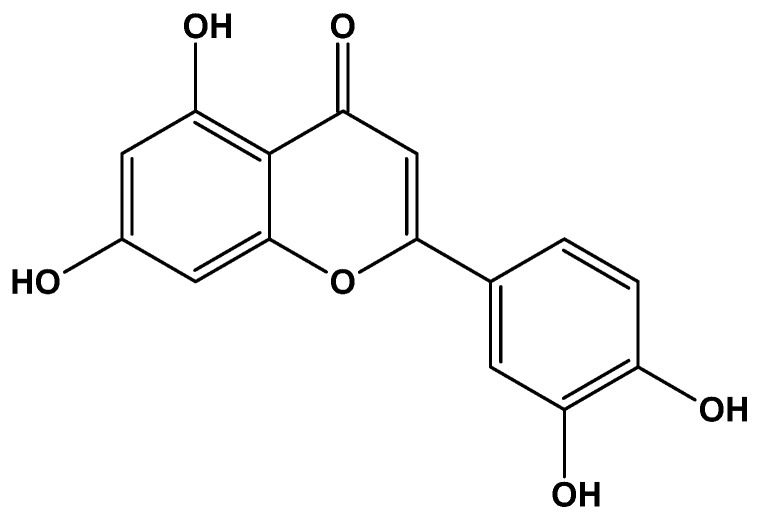
Chemical structure of luteolin.

Luteolin (3,4,5,7-tetrahydroxy flavone, Figure 1) is a natural flavonoid that is commonly found in carrots, apple, cabbage and some medicinal plants. Luteolin has displayed diverse pharmacological activities such as anti-cancer, anti-inflammatory and neuroprotective effects. Plants rich in Luteolin are used in the treatment of hypertension, inflammatory disorders, and as a preventive and therapeutic tool against different types of cancer. As illustrated in Figure 1, Luteolin possess B-ring and the 2,3-double bond in conjugation with the 4-oxo function of the C-ring. This structural propriety of Luteolin is directly associated with its anti-oxidant capacity, including the absence of oxidation during chelation with metal ions [31,32,33,34]. Studies have shown that luteolin is renoprotective against renal injury induced by different stimuli such as renal ischemia, nephrotoxic drugs and sepsis [35,36,37,38,39,40,41,42,43,44]. Moreover, luteolin has demonstrated anti-viral activities against multiple viruses, including SARS-CoV-2 [45,46,47,48,49]. Therefore, in this review, we discuss the anti-SARS-CoV-2 and renoprotective activities of luteolin. We believe that luteolin may represent a promising therapeutic for COVID-19-AKI patients.

## 2. Materials and Methods

The present article was carried out based on a survey of literature of luteolin and AKI. The search, performed in the PubMed database, included studies published until September 2022, and used the following keywords: Luteolin and COVID-19; Luteolin and SARS-CoV-2; Luteolin and acute kidney injury; Luteolin and acute kidney failure; Luteolin and SARS-CoV-2-induced acute kidney injury; Luteolin and SARS-CoV-2-induced acute renal injury; and Luteolin and COVID-19-induced acute kidney injury. Reported data of renoprotective effects of luteolin assessed by in-vitro assays or experimental models of chronic renal injury were not selected. Only studies in which the renoprotective effects of luteolin were investigated using in-vivo experimental models of AKI were selected. Results obtained from crude extract or beverages, as well as a combination of luteolin with other bioactive drugs, were not considered. Only scientific publications published in the English language were selected.

## 3. Anti-SARS-CoV-2 Activities of Luteolin

**Figure 2:**
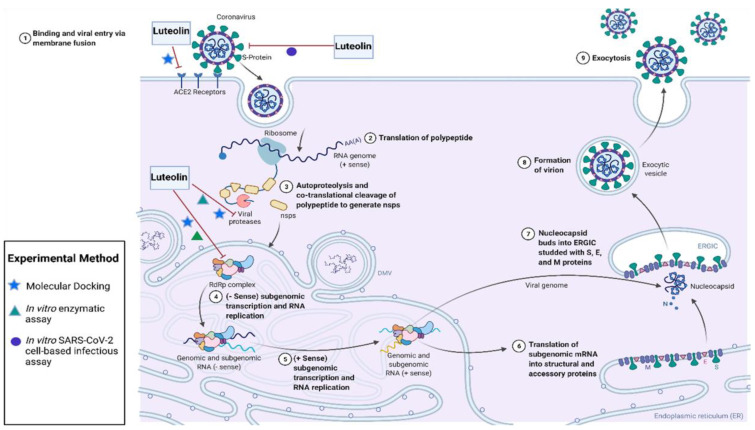
Luteolin is a potential anti-viral against SARS-CoV-2, and could be an effective therapeutic for COVID-19. Luteolin binds to S protein, and inhibits the binding of SARS-CoV-2 to ACE2. Luteolin binds with high affinity to SARS-CoV-2 main protease (Mpro), papain-like protease (PLpro), RNA-dependent RNA polymerase (RdRp) and ACE2 as shown by molecular docking studies. The inhibitory activity of luteolin for Mpro and RdRp has been confirmed by enzymatic assays. This figure was adapted from “Life Cycle of Coronavirus”, by BioRender.com (accessed on 27 January 2022), with modifications. Retrieved from https://app.biorender.com/biorender-templates (accessed on 27 January 2022).

Polyphenolic plant-derived compounds, including luteolin, have shown anti-viral activities against multiple viruses, including coronaviruses [45,46,47,50,51,52]. Since the emergence of SARS-CoV-1, several research groups have tested polyphenols, such as some flavonoids and other natural compounds for SARS-CoV-1 anti-viral activities [53]. In one study, small molecules derived from Chinese herbs were tested for binding to SARS-CoV-1 S2 domain of S protein using affinity chromatography [54]. Luteolin was identified as a compound that bound to S2 domain and inhibited the entry of both HIV-luc/SARS pseudotyped virus and SARS-CoV-1 live virus into Vero E6 cells with IC50s of 9.02 and 10 µM, respectively. Similarly, the threat of the COVID-19 pandemic has urged researchers to discover and develop effective anti-viral drugs against SARS-CoV-2. Given the efficacy of luteolin in inhibiting SARS-CoV-1 viral entry, it was tested by several research groups for anti-SARS-CoV-2 activities. Several molecular docking studies have shown that luteolin bound with high affinity to SARS-CoV-2 Mpro, PLpro, RdRP and ACE2 receptor [55,56,57,58,59,60,61,62,63,64,65,66,67]. Molecular docking studies were confirmed by fluorescence resonance energy transfer assay (FRET), which demonstrated that luteolin inhibited Mpro activity with IC50s of 11.81 µM and 20.2 µM [68,69]. Using an in-vitro enzymatic assay, luteolin was also shown to inhibit RdRp with an IC50 of 4.6  ±  0.3 µM [70]. Furthermore, co-treatment or pre-treatment of SARS-CoV-2 virus with *Vitis vinifera* leaf extract, containing derivatives of luteolin and other flavonoids, were the most effective in the inhibition of SARS-CoV-2 infection of Vero cells (80% at 10 µM) [71]. It was shown that luteolin and other extract components bound to S protein and blocked the attachment of SARS-CoV-2 to ACE2. All the previous studies demonstrate that luteolin is a promising anti-viral against SARS-CoV-2, which targets multiple viral proteins in the viral life cycle (Figure 2).

## 4. AKI: Criteria, Mechanisms and Experimental Models

AKI is defined as an abrupt and reversible decline in renal functions, which might progress to chronic kidney disease (CKD) [72]. AKI is diagnosed by criteria that include an increase in serum creatinine (SCr) ≥0.3 mg/dL (≥26.5 µmol/L) within 48 h; however, an absolute rise in SCr ≥ 2–3 times the baseline has been used as a criterion for the diagnosis and classification of advanced stages of AKI. A decrease of urinary output to less than 0.5 mL kg^−1^ h^−1^ for 6h is also used as a criterion for diagnosis of AKI [73,74]. In general, the mechanisms underlying AKI genesis are oxidative stress, inflammation and apoptosis. The oxidative stress is evidenced by a decrease in anti-oxidant enzyme activities, an increase in lipid peroxidation and elevated levels of reactive oxygen species (ROS) and reactive nitrogen species (RNS). The inflammation is characterized by a significant increase in renal expression of proinflammatory cytokines (e.g., TNF-α, IL-1β and IL-6), elevated numbers of inflammatory cells infiltrating the renal tissue and renal interstitial fibrosis. On the other hand, apoptosis is characterized by the increased expression of proapoptotic proteins (e.g., Bax) and decreased expression of antiapoptotic protein (e.g., Bcl-2) [74,75,76,77,78,79].

AKI might be experimentally induced by different stimuli, such as pharmacological models, ischemia and reperfusion (IR), sepsis and heavy metal toxicity [80,81,82,83,84].

## 5. Renoprotective Effects of Luteolin

### 5.1. Initial Considerations

Even though the present review aims to show only the experimental findings of the renoprotective effect of Lutein on AKI, its effects on CKD have already been reported and might be discussed in a further study. For example, it was observed Luteolin-containing herbal products reduced the progressive renal fibrosis induced by Unilateral ureteral obstruction (UUO), a model used for elucidating the pathogenesis of obstructive nephropathy and mechanisms responsible for progressive renal fibrosis, characterized by glomerular sclerosis and/or progressive interstitial fibrosis [85,86,87,88,89].

### 5.2. Renoprotective Effects of Luteolin against Ischemia-Reperfusion-Induced AKI

Ischemia-reperfusion (I/R) is a widely used model for clinical AKI and renal transplant studies [90,91]. The I/R model includes unilateral and bilateral renal IR [92,93,94]. I/R leads to decline of renal function, accompanied by tubular cell necrosis and apoptosis, inflammation and oxidative stress followed by increases in blood urea nitrogen (BUN) and SCr [91,92,95,96,97].

Several studies have reported that luteolin protects against I/R-induced renal injury. Hong et al. [37] reported that the oral administration of luteolin (40 mg/kg) attenuated renal histological lesions and reduced BUN and SCr levels in I/R Sprague-Dawley rats. In addition, luteolin treatment induced renal superoxide dismutase (SOD) and catalase (CAT) activities with a simultaneous reduction of renal malondialdehyde (MDA) and myeloperoxidase (MPO) levels. Moreover, serum levels of TNF-α, IL-1β and IL-6 were reduced in luteolin-treated I/R rats, which was accompanied by reduced activity of renal nuclear factor kappa B (NF-κB) [37]. In line with the previous study, Liu et al. [98] demonstrated that pre-treatment with luteolin significantly reduced the levels of TNF-α, IL-1β and IL-6, restored cellular viability of damaged renal tissue and reduced the population of apoptotic cells in I/R mice. This was accompanied with increased Bcl-2 and reduced Bax expression and reduced caspase-3 activation [98]. Furthermore, Kalbolandi et al. [99] reported that oral pre-treatment with 50 mg/kg luteolin for 3 days lowered SCr and BUN levels, attenuated renal pathological changes and increased the enzymatic activities of S OD, glutathione peroxidase (GPx) and CAT in kidneys of I/R rats. Moreover, there was a significant reduction in levels of renal tissue MDA, Nrf2 and miR320 in luteolin-pre-treated compared to untreated I/R rats [99].

### 5.3. Renoprotective Effects of Luteolin against Sepsis-Induced AKI

Sepsis is defined as a life-threatening multiorgan dysfunction caused by a dysregulated host response to infection, and is the main cause of AKI in critically-ill patients. Sepsis-induced AKI is characterized by severe inflammatory complications, high morbidity and mortality [77,100,101]. Experimental models of sepsis-induced AKI include: (1) injection of bacteria or endogenous toxins (e.g., LPS) into the peritoneum or blood; and (2) release of intestinal excreta by cecal ligation and puncture (CLP) or colon ascendens stent peritonitis (CASP) [90,102]. LPS-AKI is an acute model that is usually induced in rodents with 10–15 mg/kg LPS and terminates at 72–96 h. LPS interacts with Toll-like receptor 4 (TLR-4) on host immune cells, which induces the production of proinflammatory cytokines such as IL-1, TNF-α and IL-6, leading to inflammation [79,103,104,105]. In 2016, Xin et al. [106] showed that pre-treatment with 40 mg/kg luteolin for 3 days attenuated the LPS-induced renal damage, tubular necrosis and oxidative stress in mice. Attenuation of renal damage in luteolin pre-treated mice was supported by reduced levels of BUN and Scr and renal TNF-α, IL-1β, MCP-1, ICAM-1, NF-κB, caspase-3 [106]. The CLP model is the most frequently used model of sepsis-induced AKI, due to its simplicity, good results and reproduction of typical symptoms of bacterial peritonitis observed in humans [107,108]. Briefly, cecum is ligated from the distal to the ileocecal valve, followed by needle punctures to extrude stool into the abdominal cavity [102,107]. In a recent study by Wang et al. [42], it was observed that gastric gavage administration of 20 mg/kg/day luteoloside, 2h before the operation and for 5 days after the operation, attenuated kidney damage in CLP-induced septic mice. It was shown that luteoloside significantly reduced the production of proinflammatory cytokines, which was accompanied by inhibition of the secretion and translocation of mobility group box (HMGB)1 and HMGB1-mediated activation of TLR4/NF-κB/MAPKs signaling pathways [42].

## 6. Renoprotective Effects of Luteolin against Nephrotoxic Substances-Induced AKI

It has been reported that some compounds are potentially nephrotoxic, depending on the dose, route and duration of exposure [109,110]. For example, therapeutic drugs of different pharmacological classes (e.g., anti-cancer and antibiotics agents), heavy metals (e.g., Pb and Hg^2+^), exogenous toxins (e.g., insecticides, snake and spider poisons and industrial chemicals) and endogenous vasoactive peptides (e.g., angiotensin II) might cause renal damage with varying severity, which might range from tubular dysfunctions to severe renal failure, occasionally leading to death [111,112,113,114,115,116].

### 6.1. Renoprotective Effects of Luteolin against Glycerol-Induced AKI

Rhabdomyolysis is a syndrome in which the breakdown of skeletal muscle leads to the release of intracellular proteins and toxic compounds into circulation, which results in oxidative damage and the inflammation of different organs, including the kidneys [117,118]. AKI is considered a common complication of rhabdomyolysis, and accounts for the high mortality [119,120]. Glycerol-induced AKI is an experimental model that resembles renal injury due to rhabdomyolysis [121,122]. Oyagbemi et al. [123] reported that pre-treatment of Wistar albino rats with 100 and 200 mg/kg of luteolin for 7 days resulted in a significant reduction of oxidative stress markers, which were induced by administration of glycerol (10 mL/kg BW, 50% *v/v* in sterile saline, i.m.). Luteolin treatment attenuated the increase of renal protein carbonyl and xanthine oxidase, and increased the activities of renal GPx, glutathione S-transferase and glutathione reductase (GR). Moreover, luteolin downregulated the expression of KIM-1 and activity of NF-κB that were induced by glycerol [123].

### 6.2. Renoprotective Effects of Luteolin against Pharmacological Agents-Induced AKI

Cisplatin, Doxorubicin and Methotrexate are effective chemotherapy agents that are frequently used in the treatment of cancer and autoimmune diseases with significant renal toxicity, which limits their uses [112,124]. In rodents, the intraperitoneal (i.p.) administration of high doses of cisplatin (6–40 mg/kg), doxorubicin (2 mg/kg) or methotrexate (20 mg/kg) can induce, within 72 h, renal inflammation, oxidative stress and calcium overload, leading to significant proximal tubular toxicity, with tubular cell necrosis and apoptosis. This is usually followed by increased vascular resistance and decreased GFR, comparable with those of humans [125,126]. Domitrović et al. [35] showed that i.p. injection of luteolin (10 mg/kg), once daily for 3 days following a single cisplatin i.p. injection (10 or 20 mg/kg) in mice, significantly reduced renal dysfunction, inflammation and apoptosis induced by cisplatin. The improvement in renal function was supported by lower SCr, BUN levels and attenuated histological damages, which were accompanied by reduced renal NF-κB activity and reduced levels of TNF-α, COX-2, p53 and caspase-3 in luteolin-treated compared to untreated mice [35]. These results were in agreement with a previous study by Kang et al. [127] that showed reduced BUN, SCr, p53, PUMA-α, Bax and caspase-3 following luteolin treatment (50 mg/kg for 3 days) in cisplatin-treated C57BL/6 mice (20 mg/kg) [127]. Luteolin has also been shown to significantly improve renal function and reduce tubular cell damage, oxidative stress and apoptosis induced by doxorubicin and methotrexate. Luteolin treatment (50 and 100 mg/kg) attenuated renal dysfunction and oxidative stress induced by doxorubicin (2 mg/kg) and methotrexate (20 mg/kg) treatments. The renoprotective effect of luteolin was evidenced by lower renal MDA, ROS and RNS levels, accompanied by higher glutathione (GSH) and anti-oxidant enzyme (SOD, CAT and GPx) activities in luteolin-treated animals compared to the control group. Moreover, luteolin treatment reduced proinflammatory molecules (NF-κB, TNF-α and IL-1β) and proapoptotic proteins (Bax, caspases-3 and -9), and increased antiapoptotic (Bcl-2) proteins compared to untreated animals [43,44].

Colistin, a polymyxin antibiotic medication used as the last resort treatment for multidrug-resistant gram-negative infections, has relevant therapeutic use in clinical practice, limited by its nephrotoxicity [128,129]. In 2016, Arslan et al. [36] reported that i.p. administration of luteolin (10 mg/kg) for seven days was capable of preventing colistin-induced nephrotoxicity, as demonstrated by lowered SCr levels, and the number of apoptotic cells and renal damages compared to animals that only received colistin (480,000 IU/kg/day) [36].

### 6.3. Renoprotective Effects of Luteolin against Heavy Metals-Induced AKI

Human exposure to heavy metals; such as cobalt (Co^3+^), mercury (Hg^2+^), lead (Pb^2+^), chromium (Cr^4+^) and iron (Fe^2+^), is strongly associated with renal diseases [130,131,132]. Nitrilotriacetate (Fe-NTA) is a strong oxidant and potent nephrotoxic agent which generates highly reactive hydroxyl radical causing injuries of various organs, including kidneys. Fe-NTA-induced damage includes proximal tubular cell injury and necrosis with oxidative stress and progressive interstitial renal fibrosis [133,134]. In a study by Sultana et al. [135], the pre-treatment of Wistar rats with luteolin (10 and 20 mmol/kg) for 7 consecutive days resulted in the significant attenuation of renal lipid peroxidation and renal dysfunction induced by i.p. injection of Fe-NTA (9 mg Fe/kg). The protection of kidneys against Fe-NTA-induced damage was further demonstrated by reduced SCr and BUN, hydrogen peroxide levels, ornithine decarboxylase activity and [3H] thymidine incorporation into renal DNA [135]. Tan et al. [38] also showed that oral gavage administration of luteolin (80 mg/kg) significantly reduced HgCl_2_-induced renal damage, as shown by alleviated inflammation and oxidative stress evidenced by decreased MDA levels and NF-κB activation, as well as elevation of GSH levels. Moreover, it was observed that luteolin treatment promoted nuclear translocation of Nrf2, which was associated with increased renal expression of anti-oxidant enzymes, hemeoxygenase-1 (HO-1) and quinone-acceptor 1 (NQO1) [38]. In 2020, Oyagbemi et al. [40] reported that treatment of rats with luteolin (100 and 200 mg/kg) reversed cobalt-induced oxidative stress in kidneys by reducing renal H_2_O_2_, MDA, NO and increasing GSH, GPx and GST activities. In addition, renal tissue lesions were attenuated with reductions in serum MPO activity, renal NF-κB activity and Kim-1 expression in luteolin-treated rats compared to untreated animals [40]. Similarly, luteolin treatment protected against renal injury in Pb-treated male Wistar rats. Oral treatment with luteolin (50 mg/kg) significantly attenuated renal histological damages in Pb-treated Wistar rats (20 mg/kg, i.p), with significant reductions of SCr and BUN levels and renal MDA levels and the induction of anti-oxidant enzyme activities (SOD, CAT, GPx and GR). Additionally, luteolin inhibited the reduction in Nfe212 and Homx1 mRNA expression in Pb-treated rats, and reduced the production of proinflammatory markers (TNF-α, IL-1β and NO) and apoptotic related proteins while upregulating the expression of antiapoptotic proteins [136]. Awoyomi et al. [137] showed that the pre-treatment with Luteolin (100 and 200 mg/kg) reduced acute kidney injuries induced by potassium dichromate (K_2_Cr_2_O_7_), at dose of 30 mg/kg, through anti-oxidantive and radical scavenging mechanisms. Luteolin reduced oxidative stress indicators, augmented anti-oxidant mechanisms and serum Nitric oxide level, lowered the expressions of injury molecule (Kim-1) and up-regulated the renal, nuclear factor erythroid 2-related factor 2 (Nrf2) [137].

Bisphenol A, a nephrotoxic industrial chemical used primarily in the production of polycarbonate plastics and epoxy resins, is a recognized nephrotoxic agent with widespread daily human exposure [138]. It was shown that orally administered luteolin (100 and 200 mg/kg) protected the kidneys and increased renal Nrf2 and HO-1 expression in bisphenol-treated animals. Moreover, BUN, SCr, serum uric acid levels and proinflammatory mediators (TNF-α, IL-6 and IL-1 β) were reduced in luteolin-treated compared to untreated rats [39], Table 1.

### 6.4. Renoprotective Effects of Luteolin against Angiotensin II-Induced AKI

**Figure 3:**
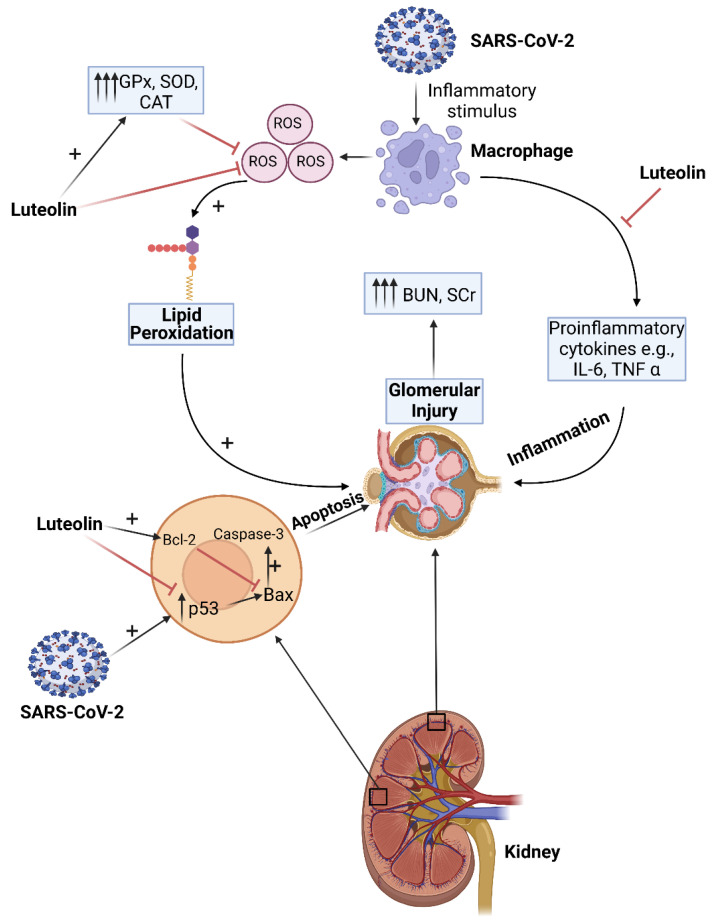
Luteolin is a potential renoprotective drug against COVID-19-associated AKI. Luteolin attenuates oxidative stress and lipid peroxidation by acting as an anti-oxidant and restoring anti-oxidant enzyme activities, which detoxify reactive oxygen species (ROS). Luteolin also acts as an anti-inflammatory by inhibiting activation of NF-kB, which lowers production of pro-inflammatory cytokines (TNF-α, IL-6 and IL-1β). Moreover, luteolin is an antiapoptotic which inhibits p53 induction and Bax expression while increasing Bcl-2 expression. This figure was created with BioRender.com (accessed on 25 January 2022).

Several studies have provided evidence of the involvement of the renin-angiotensin-aldosterone-system (RAAS) in the pathogenesis and progression of the nephropathies through renal vasoconstriction, inflammation, oxidative stress, microthrombosis and pro-proliferative effects [139,140,141,142,143]. The imbalance of RAAS is also among the suggested mechanisms of AKI development in COVID-19 patients [144,145]. Recently, Liu et al. [41] investigated the therapeutic effects of oral administration of luteolin (100 mg/kg/day for 4 weeks) on angiotensin II (AngII)-induced renal damage in apolipoprotein E-deficient (Apoe^−/−^) mice. It was reported that SCr levels and renal collagen I and III expressions were reduced in animals that were pre-treated with luteolin compared to untreated animals. Expression of IL-1β, IL-6, TNF-α and IL-10 were also suppressed in kidney tissues of the luteolin-pre-treated animals, compared to animals who received only Ang II treatment (Figure 3). Furthermore, luteolin inhibited the significant increase in LC3 protein expression, and significantly reduced p62 protein expression in kidney tissues of Ang II-treated animals [41].

## 7. Luteolin Therapeutic Effect in COVID-19-Associated AKI

The renoprotective effects of luteolin have been extensively investigated against AKI caused by different nephrotoxic stimuli (e.g., sepsis, ischemia, heavy metals and drugs) with diverse pathophysiological mechanisms [37,38,39,41,42]. Studies have shown that luteolin treatment is significantly effective in protecting against renal dysfunction induced by sepsis, ischemia and diverse nephrotoxic agents, which is demonstrated by a reduction in BUN and SCr levels, inflammatory mediators, oxidative stress and morphological damages [36,43,44]. The renoprotective effects of luteolin appear to be mostly through modulatory functions on the KIM-1/NF-κB and nuclear factor-like 2 (Nrf2)/anti-oxidant response element (ARE)/heme oxygenase 1 (HO-1) pathways [35,40,42].

Many of the pathologic features and clinical manifestations of renal injury caused by SARS-CoV-2 infection are similar to those described in kidney impairment induced by different etiologies [74,78,79]. Furthermore, glomerular and tubular damages are secondary to ischemia with redistribution of blood flow from renal medulla to the cortex, deterioration of microcirculatory oxygenation, generation of local inflammatory mediators, pro-fibrotic agents and ROS [17,18,19]. Thus, the significant renoprotective effects of luteolin in diverse experimental models of AKI, which are mediated by anti-oxidant, anti-inflammatory and antiapoptotic activities, warrant future investigation of luteolin as a potential therapeutic in COVID-19-associated AKI. Moreover, luteolin has shown renoprotective effects against AngII-induced renal damage. Since the imbalance of RAAS with generation of inflammatory mediators, oxidative stress and microthrombosis have been suggested as specific mechanisms of COVID-19-associated AKI, we believe that luteolin would be effective in managing COVID-19-associated AKI [142,146].

AKI is one of the clinical complications that represents poor prognosis, and is associated with high mortality of SARS-CoV-2-infected patients in ICU settings. The documented anti-viral and renoprotective activities of Luteolin support its further investigation as a potential drug against COVID-19-associated AKI. We believe that multiple pharmacological actions of luteolin would mitigate clinical manifestations of COVID-19, and reduce the disease progression and mortality. Luteolin can also be used as a prototype for the development of synthetic analogs with enhanced anti-inflammatory and anti-viral activities, and a better safety profile to control the current pandemic. However, two important observations must be done. Firstly, COVID-19 is a multifactorial disease, and the management of AKI with luteolin could not be the solution to improve the disease and mortality rate. Secondly, a large number of experimental and clinical studies need to be performed to ensure pharmacology safety and efficiency prior to use in the human population.

## Figures and Tables

**Table 1:** Renoprotective effects of luteolin in animal models of acute kidney injury (AKI).

**Acute Kidney Injury Induced by Ischemia**			
**Experimental Model**	**Renoprotective Effect**	**Mechanism of Action**	**Reference**
Renal ischemia-reperfusion injury	Luteolin (40 mg/kg) inhibited the increase of BUN and SCr	Reduced lipid peroxidation; Restored the depleted renal anti-oxidant enzymes (SOD, CAT and GPx); Reduced MPO activity and expression of TNF-α, IL-1β and IL-6 via suppression of NF-κB; Reduced the population of apoptotic cells with increased Bcl-2 expression accompanied by reduced Bax expression and caspase-3 activity	[37,98]
	Luteolin (50 mg/kg) inhibited the increase of BUN and SCr	Reduced the lipid peroxidation and increased glutathione levels; Restored the depleted renal SOD, CAT and GPx; Increased expression of Nrf2 and miR320	[99]
**Acute Kidney Injury Induced by Sepsis**			
**Experimental Model**	**Renoprotective Effect**	**Mechanism**	**Reference**
LPS-induced AKI	Luteolin (40 mg/kg) inhibited the increase of BUN and SCr, alleviated glomerular and tubular injury	Reduced lipid peroxidation and restored renal anti-oxidant enzymes (SOD, CAT and GPx); Decreased TNF-α, IL-1β, caspase-3, MCP-1 and ICAM-1 expression via inhibition of NF-κB,	[106]
CLP-induced AKI	Luteolin (8 and 16 mg/kg) decreased renal damages	Reduced the release of inflammatory cytokines and inhibited the secretion and translocation of mobility group box (HMGB)1 and HMGB1-mediated activation of TLR4/NF-κB/MAPKs signaling pathways	[42]
**Acute Kidney Injury Induced by nephrotoxic drugs**			
**Experimental Model**	**Renoprotective Effect**	**Mechanism**	**Reference**
Rhabdomyolysis-induced AKI (50% glycerol-10 mL/kg, i.m.)	Luteolin (100 and 200 mg/kg) inhibited the increase of BUN and SCr	Decreased renal protein carbonyl and xanthine oxidase; Increased renal glutathione; Reduced expression of KIM-1 and activity of NF-κB.	[123]
Fe-NTA-induced AKI (9 mg iron/kg, i.p.)	Luteolin (10 and 20 μmol/kg) inhibited the increase of BUN and SCr	Reduced the lipid peroxidation and increased glutathione levels; Decreased hydrogen peroxide generation, ornithine decarboxylase activity and [3H] thymidine incorporation into renal DNA promoted by Fe-NTA	[135]
Bisphenol A-induced AKI (250 mg/kg)	Luteolin (100 and 200 mg/kg) inhibited the increase of BUN and SCr	Diminished level of renal TNF-α, IL-6 and IL-1 β; increased Nrf2 and HO-1 expression	[39]
Cisplatin-induced AKI (10 and 20 mg/kg, i.p.)	Luteolin (10 mg/kg) inhibited the increase of BUN and SCr	Reduced renal activity of NF-κB, reduced expression of TNF-α, COX-2, p53 and caspase-3 activation	[35]
Potassium dichromate -induced AKI (30 mg/kg, i.p.)	Luteolin (100 and 200 mg/kg) 1	Reduced oxidative stress indicators, augmented anti-oxidant mechanisms and serum Nitric oxide level, lowered the expressions of Kim-1 and up-regulated renal Nrf2	[137]

## Data Availability

Not applicable.

## Abbreviations

AKIN	Acute Kidney Network
Akt	Protein Kinase B
AMPK	AMP-Activated Protein Kinase
ACE	angiotensin-converting enzyme
ACE 2	angiotensin-converting enzyme 2
Ang II	angiotensin II
AT1R	angiotensin type 1 receptor
Ang-(1-7)	angiotensin-(1-7)
BUN	Blood Urea Nitrogen
CAT	Catalase
GFR	Glomerular Filtration Rate
GSH-Px	Glutation Peroxidase
I/R	Ischemia And Reperfusion
ICU	Intensive Care Units
IL	Interleukin
IL-1β	Interleukin-1 Beta
IL-6	Interleukin-6
IL-18	Interleukin-18
iNOS	Nitric Oxide Synthase
INF-Υ	Interferon gamma
JNK3	C-Jun N-Terminal Kinase 3
KDIGO	Kidney Disease: Improving Global Outcomes
KIM-1	Kidney Injury Molecule-1
L-FABP	Liver-Type Fatty Acid-Binding Protein
LPS	Lipopolysaccharide
MDA	Malondialdehyde
MERS-CoV	Middle East Respiratory Syndrome-Related Coronavirus
MMP-1	Matrix Metalloproteinase-1
MyD88	Myeloid Differentiation Protein
NF-κB	Nuclear Factor Κappa B
NGAL	Neutrophil Gelatinase-Associated Lipocalin
Nrf2	Erythroid-derived 2-like 2
PI3K	Phosphoinositide 3-Kinase
RAAS	Renin-Angiotensin-Aldosterone-System
RBF	Renal Blood Flow
RIFLE	Risk, Injury, Failure, Loss, End-Stage Kidney Disease
RNS	Reactive Nitrogen Species
ROS	Reactive Oxygen Species
SCr	Serum Creatinine
SOD	Superoxide Dismutase
SYK	Tyrosine-Protein Kinase
TGF-β1	Transforming Growth Factor Beta Type I
TNF	Tumor Necrosis Factor

## References

[1] Woo P.C., Huang Y., Lau S.K., Yuen K.Y. (2010). Coronavirus genomics and bioinformatics analysis. Viruses.

[2] Woo P.C., Lau S.K., Lam C.S., Lau C.C., Tsang A.K., Lau J.H., Bai R., Teng J.L., Tsang C.C., Wang M. (2012). Discovery of seven novel Mammalian and avian coronaviruses in the genus deltacoronavirus supports bat coronaviruses as the gene source of alphacoronavirus and betacoronavirus and avian coronaviruses as the gene source of gammacoronavirus and deltacoronavirus. J. Virol..

[3] Lim Y.X., Ng Y.L., Tam J.P., Liu D.X. (2016). Human Coronaviruses: A Review of Virus-Host Interactions. Diseases.

[4] Zhu N., Zhang D., Wang W., Li X., Yang B., Song J., Zhao X., Huang B., Shi W., Lu R. (2020). China Novel Coronavirus Investigating and Research Team. A Novel Coronavirus from Patients with Pneumonia in China, 2019. N. Engl. J. Med..

[5] Drosten C., Günther S., Preiser W., van der Werf S., Brodt H.R., Becker S., Rabenau H., Panning M., Kolesnikova L., Fouchier R.A. (2003). Identification of a novel coronavirus in patients with severe acute respiratory syndrome. N. Engl. J. Med..

[6] Zaki A.M., van Boheemen S., Bestebroer T.M., Osterhaus A.D., Fouchier R.A. (2012). Isolation of a novel coronavirus from a man with pneumonia in Saudi Arabia. N. Engl. J. Med..

[7] Boban M. (2021). Novel coronavirus disease (COVID-19) update on epidemiology, pathogenicity, clinical course and treatments. Int. J. Clin. Pract..

[8] Nicola M., Alsafi Z., Sohrabi C., Kerwan A., Al-Jabir A., Iosifidis C., Agha M., Agha R. (2020). The socio-economic implications of the coronavirus pandemic (COVID-19): A review. Int. J. Surg..

[9] Hoffmann M., Kleine-Weber H., Schroeder S., Krüger N., Herrler T., Erichsen S., Schiergens T.S., Herrler G., Wu N.H., Nitsche A. (2020). SARS-CoV-2 Cell Entry Depends on ACE2 and TMPRSS2 and Is Blocked by a Clinically Proven Protease Inhibitor. Cell.

[10] Wan Y., Shang J., Graham R., Baric R.S., Li F. (2020). Receptor Recognition by the Novel Coronavirus from Wuhan: An Analysis Based on Decade-Long Structural Studies of SARS Coronavirus. J. Virol..

[11] Ou X., Liu Y., Lei X., Li P., Mi D., Ren L., Guo L., Guo R., Chen T., Hu J. (2020). Characterization of spike glycoprotein of SARS-CoV-2 on virus entry and its immune cross-reactivity with SARS-CoV. Nat. Commun..

[12] Wu A., Peng Y., Huang B., Ding X., Wang X., Niu P., Meng J., Zhu Z., Zhang Z., Wang J. (2020). Genome Composition and Divergence of the Novel Coronavirus (2019-nCoV) Originating in China. Cell Host Microbe..

[13] Gabarre P., Dumas G., Dupont T., Darmon M., Azoulay E., Zafrani L. (2020). Acute kidney injury in critically ill patients with COVID-19. Intensive Care Med..

[14] Jafari-Oori M., Fiorentino M., Castellano G., Ebadi A., Rahimi-Bashar F., Guest P.C., Vahedian-Azimi A., Sahebkar A. (2021). Acute Kidney Injury and Covid-19: A Scoping Review and Meta-Analysis. Adv. Exp. Med. Biol..

[15] Kolhe N.V., Fluck R.J., Selby N.M., Taal M.W. (2020). Acute kidney injury associated with COVID-19: A retrospective cohort study. PLoS Med..

[16] Legrand M., Bell S., Forni L., Joannidis M., Koyner J.L., Liu K., Catallupi V. (2021). Pathophysiology of COVID-19-associated acute kidney injury. Nat. Rev. Nephrol..

[17] Ugwuowo U., Yamamoto Y., Arora T., Saran I., Partridge C., Biswas A., Martin M., Moledina D.G., Greenberg J.H., Simonov M. (2020). Real-Time Prediction of Acute Kidney Injury in Hospitalized Adults: Implementation and Proof of Concept. Am. J. Kidney Dis..

[18] Barton A.L., Williams S.B.M., Dickinson S.J., Parry R.G., Pollard A. (2020). Acute Kidney Injury in Primary Care: A Review of Patient Follow-Up, Mortality, and Hospital Admissions following the Introduction of an AKI Alert System. Nephron.

[19] Chen Y.T., Shao S.C., Lai E.C., Hung M.J., Chen Y.C. (2020). Mortality rate of acute kidney injury in SARS, MERS, and COVID-19 infection: A systematic review and meta-analysis. Crit Care.

[20] Tapsell L.C., Hemphill I., Cobiac L., Patch C.S., Sullivan D.R., Fenech M., Roodenrys S., Keogh J.B., Clifton P.M., Williams P.G. (2006). Health benefits of herbs and spices: The past, the present, the future. Med. J. Aust..

[21] Meena S., Kanthaliya B., Joshi A., Khan F., Arora J. (2020). Biologia futura: Medicinal plants-derived bioactive peptides in functional perspective-a review. Biol. Futur..

[22] Wang T.Y., Li Q., Bi K.S. (2018). Bioactive flavonoids in medicinal plants: Structure, activity and biological fate. Asian J. Pharm. Sci..

[23] Gong G., Guan Y.Y., Zhang Z.L., Rahman K., Wang S.J., Zhou S., Luan X., Zhang H. (2020). Isorhamnetin: A review of pharmacological effects. Biomed. Pharmacother..

[24] Batiha G.E., Beshbishy A.M., Ikram M., Mulla Z.S., El-Hack M.E.A., Taha A.E., Algammal A.M., Elewa Y.H.A. (2020). The Pharmacological Activity, Biochemical Properties, and Pharmacokinetics of the Major Natural Polyphenolic Flavonoid: Quercetin. Foods.

[25] Negri A., Naponelli V., Rizzi F., Bettuzzi S. (2018). Molecular Targets of Epigallocatechin-Gallate (EGCG): A Special Focus on Signal Transduction and Cancer. Nutrients.

[26] Gupta G., Siddiqui M.A., Khan M.M., Ajmal M., Ahsan R., Rahaman M.A., Ahmad M.A., Arshad M., Khushtar M. (2020). Current Pharmacological Trends on Myricetin. Drug Res..

[27] Lam K.Y., Ling A.P., Koh R.Y., Wong Y.P., Say Y.H. (2016). A Review on Medicinal Properties of Orientin. Adv. Pharmacol. Sci..

[28] Procházková D., Boušová I., Wilhelmová N. (2011). Antioxidant and prooxidant properties of flavonoids. Fitoterapia.

[29] Wu S., Zhang Y., Ren F., Qin Y., Liu J., Liu J., Wang Q., Zhang H. (2018). Structure-affinity relationship of the interaction between phenolic acids and their derivatives and β-lactoglobulin and effect on antioxidant activity. Food Chem..

[30] Diniz L.R.L., Bezerra Filho C.D.S.M., Fielding B.C., de Sousa D.P. (2020). Natural Antioxidants: A Review of Studies on Human and Animal Coronavirus. Oxid. Med. Cell Longev..

[31] Lin Y., Shi R., Wang X., Shen H.M. (2008). Luteolin, a flavonoid with potential for cancer prevention and therapy. Curr. Cancer Drug Targets.

[32] Nabavi S.F., Braidy N., Gortzi O., Sobarzo-Sanchez E., Daglia M., Skalicka-Woźniak K., Nabavi S.M. (2015). Luteolin as an anti-inflammatory and neuroprotective agent: A brief review. Brain Res. Bull..

[33] Imran M., Rauf A., Abu-Izneid T., Nadeem M., Shariati M.A., Khan I.A., Imran A., Orhan I.E., Rizwan M., Atif M. (2019). Luteolin, a flavonoid, as an anticancer agent: A review. Biomed. Pharmacother..

[34] Manzoor M.F., Ahmad N., Ahmed Z., Siddique R., Zeng X.A., Rahaman A., Muhammad Aadil R., Wahab A. (2019). Novel extraction techniques and pharmaceutical activities of luteolin and its derivatives. J. Food Biochem..

[35] Domitrović R., Cvijanović O., Pugel E.P., Zagorac G.B., Mahmutefendić H., Škoda M. (2013). Luteolin ameliorates cisplatin-induced nephrotoxicity in mice through inhibition of platinum accumulation, inflammation and apoptosis in the kidney. Toxicology.

[36] Arslan B.Y., Arslan F., Erkalp K., Alagöl A., Sevdi M.S., Yıldız G., Küçük S.H., Altınay S. (2016). Luteolin ameliorates colistin-induced nephrotoxicity in the rat models. Ren. Fail..

[37] Hong X., Zhao X., Wang G., Zhang Z., Pei H., Liu Z. (2017). Luteolin Treatment Protects against Renal Ischemia-Reperfusion Injury in Rats. Mediat. Inflamm..

[38] Tan X., Liu B., Lu J., Li S., Baiyun R., Lv Y., Lu Q., Zhang Z. (2018). Dietary luteolin protects against Hg2+Cl2-induced renal injury via activation of Nrf2-mediated signaling in rat. J. Inorg. Biochem..

[39] Alekhya Sita G.J., Gowthami M., Srikanth G., Krishna M.M., Rama Sireesha K., Sajjarao M., Nagarjuna K., Nagarjuna M., Chinnaboina G.K., Mishra A. (2019). Protective role of luteolin against bisphenol A-induced renal toxicity through suppressing oxidative stress, inflammation, and upregulating Nrf2/ARE/ HO-1 pathway. IUBMB Life.

[40] Oyagbemi A.A., Akinrinde A.S., Adebiyi O.E., Jarikre T.A., Omobowale T.O., Ola-Davies O.E., Saba A.B., Emikpe B.O., Adedapo A.A. (2020). Luteolin supplementation ameliorates cobalt-induced oxidative stress and inflammation by suppressing NF-кB/Kim-1 signaling in the heart and kidney of rats. Environ. Toxicol. Pharmacol..

[41] Liu Y.S., Yang Q., Li S., Luo L., Liu H.Y., Li X.Y., Gao Z.N. (2021). Luteolin attenuates angiotensin II-induced renal damage in apolipoprotein E-deficient mice. Mol. Med. Rep..

[42] Wang Z., Chen W., Li Y., Zhang S., Lou H., Lu X., Fan X. (2021). Reduning injection and its effective constituent luteoloside protect against sepsis partly via inhibition of HMGB1/TLR4/NF-κB/MAPKs signaling pathways. J. Ethnopharmacol..

[43] Owumi S.E., Lewu D.O., Arunsi U.O., Oyelere A.K. (2021). Luteolin attenuates doxorubicin-induced derangements of liver and kidney by reducing oxidative and inflammatory stress to suppress apoptosis. Hum. Exp. Toxicol..

[44] Dar A.A., Fehaid A., Alkhatani S., Alarifi S., Alqahtani W.S., Albasher G., Almeer R., Alfarraj S., Moneim A.A. (2021). The protective role of luteolin against the methotrexate-induced hepato-renal toxicity via its antioxidative, anti-inflammatory, and anti-apoptotic effects in rats. Hum. Exp. Toxicol..

[45] Cui X.X., Yang X., Wang H.J., Rong X.Y., Jing S., Xie Y.H., Huang D.F., Zhao C. (2017). Luteolin-7-O-Glucoside Present in Lettuce Extracts Inhibits Hepatitis B Surface Antigen Production and Viral Replication by Human Hepatoma Cells in Vitro. Front. Microbiol..

[46] Xu L., Su W., Jin J., Chen J., Li X., Zhang X., Sun M., Sun S., Fan P., An D. (2014). Identification of luteolin as enterovirus 71 and coxsackievirus A16 inhibitors through reporter viruses and cell viability-based screening. Viruses.

[47] Ayipo Y.O., Yahaya S.N., Alananzeh W.A., Babamale H.F., Mordi M.N. (2021). Pathomechanisms, therapeutic targets and potent inhibitors of some beta-coronaviruses from bench-to-bedside. Infect Genet Evol..

[48] Wang S., Ling Y., Yao Y., Zheng G., Chen W. (2020). Luteolin inhibits respiratory syncytial virus replication by regulating the MiR-155/SOCS1/STAT1 signaling pathway. Virol. J..

[49] Chiou W.C., Lu H.F., Hsu N.Y., Chang T.Y., Chin Y.F., Liu P.C., Lo J.M., Wu Y.B., Yang J.M., Huang C. (2021). Ugonin J Acts as a SARS-CoV-2 3C-like Protease Inhibitor and Exhibits Anti-inflammatory Properties. Front. Pharmacol..

[50] Pusztai R., Béládi I., Bakai M., Mucsi I., Kukán E. (1966). Study on the effect of flavonoids and related substances. I. The effect of quercetin on different viruses. Acta Microbiol. Acad. Sci. Hung..

[51] Kaul T.N., Middleton E., Ogra P.L. (1985). Antiviral effect of flavonoids on human viruses. J. Med. Virol..

[52] Zhang W., Qiao H., Lv Y., Wang J., Chen X., Hou Y., Tan R., Li E. (2014). Apigenin inhibits enterovirus-71 infection by disrupting viral RNA association with trans-acting factors. PLoS ONE..

[53] Elshabrawy H.A. (2020). SARS-CoV-2: An Update on Potential Antivirals in Light of SARS-CoV Antiviral Drug Discoveries. Vaccines.

[54] Yi L., Li Z., Yuan K., Qu X., Chen J., Wang G., Zhang H., Luo H., Zhu L., Jiang P. (2004). Small molecules blocking the entry of severe acute respiratory syndrome coronavirus into host cells. J. Virol..

[55] Yalçın S., Yalçınkaya S., Ercan F. (2021). *In silico* detection of inhibitor potential of *Passiflora* compounds against SARS-CoV-2(Covid-19) main protease by using molecular docking and dynamic analyses. J. Mol. Struct..

[56] Vincent S., Arokiyaraj S., Saravanan M., Dhanraj M. (2020). Molecular Docking Studies on the Anti-viral Effects of Compounds From Kabasura Kudineer on SARS-CoV-2 3CLpro. Front. Mol. Biosci..

[57] Mohapatra P.K., Chopdar K.S., Dash G.C., Mohanty A.K., Raval M.K. (2021). In silico screening and covalent binding of phytochemicals of Ocimum sanctum against SARS-CoV-2 (COVID 19) main protease. J. Biomol. Struct. Dyn..

[58] Alamri M.A., Altharawi A., Alabbas A.B., Alossaimi M.A., Alqahtani S.M. (2020). Structure-based virtual screening and molecular dynamics of phytochemicals derived from Saudi medicinal plants to identify potential COVID-19 therapeutics. Arab. J. Chem..

[59] Zhang X., Gao R., Zhou Z., Tang X., Lin J., Wang L., Zhou X., Shen T. (2021). A network pharmacology based approach for predicting active ingredients and potential mechanism of Lianhuaqingwen capsule in treating COVID-19. Int. J. Med. Sci..

[60] Gyebi G.A., Elfiky A.A., Ogunyemi O.M., Ibrahim I.M., Adegunloye A.P., Adebayo J.O., Olaiya C.O., Ocheje J.O., Fabusiwa M.M. (2021). Structure-based virtual screening suggests inhibitors of 3-Chymotrypsin-Like Protease of SARS-CoV-2 from *Vernonia amygdalina* and *Occinum gratissimum*. Comput. Biol. Med..

[61] Xia S., Zhong Z., Gao B., Vong C.T., Lin X., Cai J., Gao H., Chan G., Li C. (2021). The important herbal pair for the treatment of COVID-19 and its possible mechanisms. Chin. Med..

[62] Nouadi B., Ezaouine A., El Messal M., Blaghen M., Bennis F., Chegdani F. (2021). Prediction of Anti-COVID 19 Therapeutic Power of Medicinal Moroccan Plants Using Molecular Docking. Bioinform. Biol. Insights.

[63] Maurya V.K., Kumar S., Prasad A.K., Bhatt M.L.B., Saxena S.K. (2020). Structure-based drug designing for potential antiviral activity of selected natural products from Ayurveda against SARS-CoV-2 spike glycoprotein and its cellular receptor. Virusdisease.

[64] Rakshit G., Dagur P., Satpathy S., Patra A., Jain A., Ghosh M. (2022). Flavonoids as potential therapeutics against novel coronavirus disease-2019 (nCOVID-19). J. Biomol. Struct. Dyn..

[65] Ye X.W., Deng Y.L., Zhang X., Liu M.M., Liu Y., Xie Y.T., Wan Q., Huang M., Zhang T., Xi J.H. (2021). Study on the Mechanism of treating COVID-19 with Shenqi Wan based on Network Pharmacology. Drug Dev. Ind. Pharm..

[66] Kumar B., Zaidi S., Haque S., Dasgupta N., Hussain A., Pramodh S., Singh V., Mishra B.N. (2021). In Silico Studies Reveal Antiviral Effects of Traditional Indian Spices on COVID-19. Curr. Pharm. Des..

[67] Yu R., Chen L., Lan R., Shen R., Li P. (2020). Computational screening of antagonists against the SARS-CoV-2 (COVID-19) coronavirus by molecular docking. Int. J. Antimicrob. Agents.

[68] Ryu Y.B., Jeong H.J., Kim J.H., Kim Y.M., Park J.Y., Kim D., Nguyen T.T., Park S.J., Chang J.S., Park K.H. (2010). Biflavonoids from *Torreya nucifera* displaying SARS-CoV 3CL(pro) inhibition. Bioorg. Med. Chem..

[69] Shahhamzehei N., Abdelfatah S., Efferth T. (2022). In Silico and In Vitro Identification of Pan-Coronaviral Main Protease Inhibitors from a Large Natural Product Library. Pharmaceuticals.

[70] Munafò F., Donati E., Brindani N., Ottonello G., Armirotti A., De Vivo M. (2022). Quercetin and luteolin are single-digit micromolar inhibitors of the SARS-CoV-2 RNA-dependent RNA polymerase. Sci. Rep..

[71] Zannella C., Giugliano R., Chianese A., Buonocore C., Vitale G.A., Sanna G., Sarno F., Manzin A., Nebbioso A., Termolino P. (2021). Antiviral Activity of *Vitis vinifera* Leaf Extract against SARS-CoV-2 and HSV-1. Viruses.

[72] Rossaint J., Zarbock A. (2016). Acute kidney injury: Definition, diagnosis and epidemiology. Minerva Urol. Nefrol..

[73] Koza Y. (2016). Acute kidney injury: Current concepts and new insights. J. Inj. Violence Res..

[74] Ronco C., Bellomo R., Kellum J.A. (2019). Acute kidney injury. Lancet.

[75] Sharfuddin A.A., Molitoris B.A. (2011). Pathophysiology of ischemic acute kidney injury. Nat. Rev. Nephrol..

[76] Basile D.P., Anderson M.D., Sutton T.A. (2012). Pathophysiology of acute kidney injury. Compr. Physiol..

[77] Peerapornratana S., Manrique-Caballero C.L., Gómez H., Kellum J.A. (2019). Acute kidney injury from sepsis: Current concepts, epidemiology, pathophysiology, prevention and treatment. Kidney Int..

[78] Bansal S., Patel R.N. (2020). Pathophysiology of Contrast-Induced Acute Kidney Injury. Interv. Cardiol. Clin..

[79] Manrique-Caballero C.L., Del Rio-Pertuz G., Gomez H. (2021). Sepsis-Associated Acute Kidney Injury. Crit Care Clin..

[80] Heyman S.N., Rosenberger C., Rosen S. (2011). Acute kidney injury: Lessons from experimental models. Contrib. Nephrol..

[81] Bao Y.W., Yuan Y., Chen J.H., Lin W.Q. (2018). Kidney disease models: Tools to identify mechanisms and potential therapeutic targets. Zool Res..

[82] Fu Y., Tang C., Cai J., Chen G., Zhang D., Dong Z. (2018). Rodent models of AKI-CKD transition. Am. J. Physiol. Renal. Physiol..

[83] Neyra J.A., Leaf D.E. (2018). Risk Prediction Models for Acute Kidney Injury in Critically Ill Patients: Opus in Progressu. Nephron.

[84] Shiva N., Sharma N., Kulkarni Y.A., Mulay S.R., Gaikwad A.B. (2020). Renal ischemia/reperfusion injury: An insight on in vitro and in vivo models. Life Sci..

[85] Ucero A.C., Benito-Martin A., Izquierdo M.C., Sanchez-Niño M.D., Sanz A.B., Ramos A.M., Berzal S., Ruiz-Ortega M., Egido J., Ortiz A. (2014). Unilateral ureteral obstruction: Beyond obstruction. Int. Urol. Nephrol..

[86] Narváez Barros A., Guiteras R., Sola A., Manonelles A., Morote J., Cruzado J.M. (2019). Reversal Unilateral Ureteral Obstruction: A Mice Experimental Model. Nephron.

[87] Chevalier R.L., Forbes M.S., Thornhill B.A. (2009). Ureteral obstruction as a model of renal interstitial fibrosis and obstructive nephropathy. Kidney Int..

[88] Song J., Liu J., Luo J., Zhang Q., Xia Y., Shao Q., Sun C., Jiang C., Zhang M., Zhu W. (2019). A modified relief of unilateral ureteral obstruction model. Ren. Fail..

[89] Kim T.W., Kim Y.J., Seo C.S., Kim H.T., Park S.R., Lee M.Y., Jung J.Y. (2016). *Elsholtzia ciliata* (Thunb.) Hylander attenuates renal inflammation and interstitial fibrosis via regulation of TGF-ß and Smad3 expression on unilateral ureteral obstruction rat model. Phytomedicine.

[90] Singh A.P., Junemann A., Muthuraman A., Jaggi A.S., Singh N., Grover K., Dhawan R. (2012). Animal models of acute renal failure. Pharmacol. Rep..

[91] Faucher Q., Alarcan H., Marquet P., Barin-Le Guellec C. (2020). Effects of Ischemia-Reperfusion on Tubular Cell Membrane Transporters and Consequences in Kidney Transplantation. J. Clin. Med..

[92] Hesketh E.E., Czopek A., Clay M., Borthwick G., Ferenbach D., Kluth D., Hughes J. (2014). Renal ischaemia reperfusion injury: A mouse model of injury and regeneration. J. Vis. Exp..

[93] Packialakshmi B., Stewart I.J., Burmeister D.M., Chung K.K., Zhou X. (2020). Large animal models for translational research in acute kidney injury. Ren. Fail..

[94] Zhang J., Wang X., Wei J., Wang L., Jiang S., Xu L., Qu L., Yang K., Fu L., Buggs J. (2020). A two-stage bilateral ischemia-reperfusion injury-induced AKI to CKD transition model in mice. Am. J. Physiol. Renal. Physiol..

[95] Black L.M., Lever J.M., Traylor A.M., Chen B., Yang Z., Esman S.K., Jiang Y., Cutter G.R., Boddu R., George J.F. (2018). Divergent effects of AKI to CKD models on inflammation and fibrosis. Am. J. Physiol. Renal. Physiol..

[96] Banaei S., Rezagholizadeh L. (2019). The role of hormones in renal disease and ischemia-reperfusion injury. Iran J. Basic Med. Sci..

[97] Maeda A., Hayase N., Doi K. (2020). Acute Kidney Injury Induces Innate Immune Response and Neutrophil Activation in the Lung. Front. Med..

[98] Liu Y., Shi B., Li Y., Zhang H. (2017). Protective Effect of Luteolin Against Renal Ischemia/Reperfusion Injury via Modulation of Pro-Inflammatory Cytokines, Oxidative Stress and Apoptosis for Possible Benefit in Kidney Transplant. Med. Sci. Monit..

[99] Kalbolandi S.M., Gorji A.V., Babaahmadi-Rezaei H., Mansouri E. (2019). Luteolin confers renoprotection against ischemia-reperfusion injury via involving Nrf2 pathway and regulating miR320. Mol. Biol. Rep..

[100] Bellomo R., Kellum J.A., Ronco C., Wald R., Martensson J., Maiden M., Bagshaw S.M., Glassford N.J., Lankadeva Y., Vaara S.T. (2017). Acute kidney injury in sepsis. Intensive Care Med..

[101] Poston J.T., Koyner J.L. (2019). Sepsis associated acute kidney injury. BMJ.

[102] Doi K., Leelahavanichkul A., Yuen P.S., Star R.A. (2009). Animal models of sepsis and sepsis-induced kidney injury. J. Clin. Invest..

[103] Vorobeva E.V., Krasikova I.N., Solov’eva T.F. (2006). Influence of lipopolysaccharides and lipids A from some marine bacteria on spontaneous and Escherichia coli LPS-induced TNF-alpha release from peripheral human blood cells. Biochemistry.

[104] Solov’eva T., Davydova V., Krasikova I., Yermak I. (2013). Marine compounds with therapeutic potential in gram-negative sepsis. Mar. Drugs.

[105] Yoo J.Y., Cha D.R., Kim B., An E.J., Lee S.R., Cha J.J., Kang Y.S., Ghee J.Y., Han J.Y., Bae Y.S. (2020). LPS-Induced Acute Kidney Injury Is Mediated by Nox4-SH3YL1. Cell Rep..

[106] Xin S.B., Yan H., Ma J., Sun Q., Shen L. (2016). Protective Effects of Luteolin on Lipopolysaccharide-Induced Acute Renal Injury in Mice. Med. Sci. Monit..

[107] Dejager L., Pinheiro I., Dejonckheere E., Libert C. (2011). Cecal ligation and puncture: The gold standard model for polymicrobial sepsis?. Trends Microbiol..

[108] Li J.L., Li G., Jing X.Z., Li Y.F., Ye Q.Y., Jia H.H., Liu S.H., Li X.J., Li H., Huang R. (2018). Assessment of clinical sepsis-associated biomarkers in a septic mouse model. J. Int. Med. Res..

[109] Perazella M.A. (2018). Pharmacology behind Common Drug Nephrotoxicities. Clin. J. Am. Soc. Nephrol..

[110] Wu H., Huang J. (2018). Drug-Induced Nephrotoxicity: Pathogenic Mechanisms, Biomarkers and Prevention Strategies. Curr. Drug Metab..

[111] Madden E.F., Fowler B.A. (2000). Mechanisms of nephrotoxicity from metal combinations: A review. Drug Chem. Toxicol..

[112] Malyszko J., Kozlowska K., Kozlowski L., Malyszko J. (2017). Nephrotoxicity of anticancer treatment. Nephrol. Dial. Transplant..

[113] McWilliam S.J., Antoine D.J., Smyth R.L., Pirmohamed M. (2017). Aminoglycoside-induced nephrotoxicity in children. Pediatr. Nephrol..

[114] Barnett L.M.A., Cummings B.S. (2018). Nephrotoxicity and Renal Pathophysiology: A Contemporary Perspective. Toxicol. Sci..

[115] Chou Y.H., Chu T.S., Lin S.L. (2018). Role of renin-angiotensin system in acute kidney injury-chronic kidney disease transition. Nephrology.

[116] Leowattana W. (2019). Antiviral Drugs and Acute Kidney Injury (AKI). Infect Disorder. Drug Targets.

[117] Chavez L.O., Leon M., Einav S., Varon J. (2016). Beyond muscle destruction: A systematic review of rhabdomyolysis for clinical practice. Crit. Care.

[118] Cabral B.M.I., Edding S.N., Portocarrero J.P., Lerma E.V. (2020). Rhabdomyolysis. Dis. Mon..

[119] Bosch X., Poch E., Grau J.M. (2009). Rhabdomyolysis and acute kidney injury. N. Engl. J. Med..

[120] Petejova N., Martinek A. (2014). Acute kidney injury due to rhabdomyolysis and renal replacement therapy: A critical review. Crit. Care.

[121] Geng Y., Zhang L., Fu B., Zhang J., Hong Q., Hu J., Li D., Luo C., Cui S., Zhu F. (2014). Mesenchymal stem cells ameliorate rhabdomyolysis-induced acute kidney injury via the activation of M2 macrophages. Stem. Cell Res. Ther..

[122] Kim J.Y., Bai Y., Jayne L.A., Cianciolo R.E., Bajwa A., Pabla N.S. (2020). Involvement of the CDKL5-SOX9 signaling axis in rhabdomyolysis-associated acute kidney injury. Am. J. Physiol. Renal. Physiol..

[123] Oyagbemi A.A., Adejumobi O.A., Ajibade T.O., Asenuga E.R., Afolabi J.M., Ogunpolu B.S., Falayi O.O., Hassan F.O., Nabofa E.W., Olutayo Omobowale T. (2021). Luteolin Attenuates Glycerol-Induced Acute Renal Failure and Cardiac Complications Through Modulation of Kim-1/NF-κB/Nrf2 Signaling Pathways. J. Diet Suppl..

[124] Izzedine H., Perazella M.A. (2017). Anticancer Drug-Induced Acute Kidney Injury. Kidney Int. Rep..

[125] Perazella M.A. (2019). Drug-induced acute kidney injury: Diverse mechanisms of tubular injury. Curr. Opin. Crit. Care.

[126] Santos M.L.C., de Brito B.B., da Silva F.A.F., Botelho A.C.D.S., de Melo F.F. (2020). Nephrotoxicity in cancer treatment: An overview. World J. Clin. Oncol..

[127] Kang K.P., Park S.K., Kim D.H., Sung M.J., Jung Y.J., Lee A.S., Lee J.E., Ramkumar K.M., Lee S., Park M.H. (2016). Luteolin ameliorates cisplatin-induced acute kidney injury in mice by regulation of p53-dependent renal tubular apoptosis. Nephrol. Dial. Transplant.

[128] Heybeli C., Oktan M.A., Çavdar Z. (2019). Rat models of colistin nephrotoxicity: Previous experimental researches and future perspectives. Eur. J. Clin. Microbiol. Infect Dis..

[129] Gunay E., Kaya S., Baysal B., Yuksel E., Arac E. (2020). Evaluation of prognosis and nephrotoxicity in patients treated with colistin in intensive care unit. Ren. Fail..

[130] Van Vleet T.R., Schnellmann R.G. (2003). Toxic nephropathy: Environmental chemicals. Semin. Nephrol..

[131] Sabath E., Robles-Osorio M.L. (2012). Renal health and the environment: Heavy metal nephrotoxicity. Nefrologia.

[132] Riaz M.A., Nisa Z.U., Mehmood A., Anjum M.S., Shahzad K. (2019). Metal-induced nephrotoxicity to diabetic and non-diabetic Wistar rats. Environ. Sci. Pollut. Res. Int..

[133] Chopra K., Singh D., Chander V. (2004). Nephrotoxicity and its prevention by catechin in ferric nitrilotriacetate promoted oxidative stress in rats. Hum. Exp. Toxicol..

[134] Eybl V., Kotyzová D., Cerná P., Koutensky J. (2008). Effect of melatonin, curcumin, quercetin, and resveratrol on acute ferric nitrilotriacetate (Fe-NTA)-induced renal oxidative damage in rats. Hum. Exp. Toxicol..

[135] Sultana S., Prasad L., Jahangir T. (2009). Luteolin ameliorates ferric nitrilotriacetic acid induced renal toxicity and tumor promotional response in rat. Indian J. Exp. Biol..

[136] Albarakati A.J.A., Baty R.S., Aljoudi A.M., Habotta O.A., Elmahallawy E.K., Kassab R.B., Abdel Moneim A.E. (2020). Luteolin protects against lead acetate-induced nephrotoxicity through antioxidant, anti-inflammatory, anti-apoptotic, and Nrf2/HO-1 signaling pathways. Mol. Biol. Rep..

[137] Awoyomi O.V., Adeoye Y.D., Oyagbemi A.A., Ajibade T.O., Asenuga E.R., Gbadamosi I.T., Ogunpolu B.S., Falayi O.O., Hassan F.O., Omobowale T.O. (2021). Luteolin mitigates potassium dichromate-induced nephrotoxicity, cardiotoxicity and genotoxicity through modulation of Kim-1/Nrf2 signaling pathways. Environ. Toxicol..

[138] Kobroob A., Peerapanyasut W., Chattipakorn N., Wongmekiat O. (2018). Damaging Effects of Bisphenol A on the Kidney and the Protection by Melatonin: Emerging Evidences from In Vivo and In Vitro Studies. Oxid. Med. Cell Longev..

[139] Michel M.C., Brunner H.R., Foster C., Huo Y. (2016). Angiotensin II type 1 receptor antagonists in animal models of vascular, cardiac, metabolic and renal disease. Pharmacol. Ther..

[140] Remuzzi A., Sangalli F., Macconi D., Tomasoni S., Cattaneo I., Rizzo P., Bonandrini B., Bresciani E., Longaretti L., Gagliardini E. (2016). Regression of Renal Disease by Angiotensin II Antagonism Is Caused by Regeneration of Kidney Vasculature. J. Am. Soc. Nephrol..

[141] Hsu C.Y., Liu K.D., Yang J., Glidden D.V., Tan T.C., Pravoverov L., Zheng S., Go A.S. (2020). Renin-Angiotensin System Blockade after Acute Kidney Injury (AKI) and Risk of Recurrent AKI. Clin. J. Am. Soc. Nephrol..

[142] Hines A., Li X., Ortiz-Soriano V., Saleh S., Litteral J., Ruiz-Conejo M., Wald R., Silver S.A., Neyra J.A. (2020). Use of Angiotensin-Converting Enzyme Inhibitors/Angiotensin Receptor Blockers and Acute Kidney Disease after an Episode of AKI: A Multicenter Prospective Cohort Study. Am. J. Nephrol..

[143] Siew E.D., Parr S.K., Abdel-Kader K., Perkins A.M., Greevy R.A., Vincz A.J., Denton J., Wilson O.D., Hung A.M., Ikizler T.A. (2021). Renin-angiotensin aldosterone inhibitor use at hospital discharge among patients with moderate to severe acute kidney injury and its association with recurrent acute kidney injury and mortality. Kidney Int..

[144] Simões E., Silva A.C., Lanza K., Palmeira V.A., Costa L.B., Flynn J.T. (2021). 2020 update on the renin-angiotensin-aldosterone system in pediatric kidney disease and its interactions with coronavirus. Pediatr. Nephrol..

[145] Dudoignon E., Moreno N., Deniau B., Coutrot M., Longer R., Amiot Q., Mebazaa A., Pirracchio R., Depret F., Legrand M. (2020). Activation of the renin-angiotensin-aldosterone system is associated with Acute Kidney Injury in COVID-19. Anaesth. Crit. Care Pain Med..

[146] Soleimani M. (2020). Acute Kidney Injury in SARS-CoV-2 Infection: Direct Effect of Virus on Kidney Proximal Tubule Cells. Int. J. Mol. Sci..

